# Safety and Efficacy of Procedural Sedation and Analgesia in Pediatric Oncology Patients

**DOI:** 10.7759/cureus.7442

**Published:** 2020-03-28

**Authors:** Saba Laila Aslam, Anwar Haque, Muhammad Tariq Jamil, Madiha Ariff, Saad Nasir

**Affiliations:** 1 Pediatrics, The Indus Hospital, Karachi, PAK; 2 Pediatrics, Liaquat National Hospital, Karachi, PAK; 3 Internal Medicine, Dow University of Health Sciences, Karachi, PAK; 4 Internal Medicine, United Medical and Dental College, Creek General Hospital, Karachi, PAK

**Keywords:** procedural sedation and analgesia, safety, efficacy, ketamine, propofol

## Abstract

Objective

Procedural sedation and analgesia are the standard of care for painful procedures in children that require immobility. The aim is to assess the safety and efficacy of procedural sedation and analgesia in pediatric oncological patients in a large tertiary care hospital.

Method

An observational study performed to review medical records of children who received procedural sedation and analgesia (PSA) for pediatric oncological procedures from July 2018 to September 2018. Patients undergoing oncology procedures (lumbar puncture, intrathecal chemotherapy, bone marrow aspiration +/- trephine) were included, and non-anesthesiologist (intensive care physician/emergency physician certified in pediatric advanced life support) provided PSA. Patients were assessed according to PSA protocol guidelines by the American Society of Anesthesiology (ASA). Low-dose ketamine (0.5 mg/kg) and propofol (2 mg/kg) were administered.

Results

A total of 565 children underwent 1216 procedures in whom the median age was 7.4 years, and the majority (65.1%) were males. The most common procedure was the lumbar puncture (n = 956; 78.6%) followed by bone marrow aspirate only (n = 137, 11.3%) and both (n = 123, 10.1%). Eight (0.7%) patients developed transient oxygen desaturation only as an adverse effect of ketamine-propofol drug combination with 50% procedures utilizing propofol 1 mg/kg for sedation.

Conclusion

According to the results of our study, the majority of the pediatric patients responded and reported no adverse events during the procedure with ketamine and propofol. Therefore, we conclude that ketamine and propofol are safe and effective as both sedative and an analgesic in procedures on pediatric oncology patients.

## Introduction

Procedural sedation and analgesia (PSA) are the standard of care for painful procedures in children that require immobility [1]. Children with cancer undergo many procedures during the treatment, which are often painful and cause significant anxiety in them. Procedural sedation provides safe and effective control of pain and anxiety, which leads to an adequate degree of memory loss and minimal awareness of the procedure [2]. There are guidelines proposed by the American Academy of Pediatrics (AAP) and Joint Commission International Accreditation (JCIA) regarding the management of pain and anxiety associated with these procedures in children [3-4].

Anesthetists, physicians, intensivists, or emergency physicians usually administer procedural sedation [5]. The main aim of intravenous sedation for procedural anxiety is the establishment of a state of relaxation with intact protective reflexes [6]. Children who undergo procedures in such a state of relaxation and comfort are less anxious and fearful during, after and before a planned procedure [7].

Relief from anxiety and procedural pain is ethically imperative in treating children from the fact that they tend to have short- and long-term physiological, physical as well as a psychological effect because of untreated pain [8]. Therefore, procedural sedation, described as the administration of medication for minimizing pain and awareness of the patients, is termed as the standard practice in the pediatric department’s world over for facilitating procedures [9]. Although sedation is usually safe, it is sometimes associated with serious adverse events (SAEs). The prevalence of SAEs is difficult to determine since their occurrence is infrequent, and due to the lack of large multicentered studies, which focus on the systemic findings of the adverse events [10]. A serious adverse event (SAE) may include apnea, hypotension, laryngospasm, bradycardia, clinically apparent pulmonary aspiration, complete airway obstruction, and permanent neurological damage or even death. The interventions performed in response to SAEs such as positive pressure ventilation, administration of vasoactive or neuromuscular blockade drugs, and endotracheal intubation or chest compressions are considered substantial [11].

There are several ways to determine the risk factors for the occurrence of SAE in pediatric patients. From the basis of prior clinical knowledge, reported adverse events of the sedative drugs must be kept in mind [12]. Other factors may include age, gender, and body mass index, underlying health risks (health issues which might affect sedation efficacy or adverse event incidence), any on-going respiratory illness, administration of opioids before a procedure, fasting for solids for 4 to 6 hours and liquids for about 2 hours, type of procedure, duration of the procedure and procedural complications. Pre-procedural risk factors must be determined through patients or parent reports, reviewing medical records, and by findings of general physical examination [13].

An essential aspect of the safety and efficacy of procedural sedation and analgesia is the choosing or combination of drugs to be used for sedating the child. Various factors should be taken into account before ensuring tolerability, successful sedation, and facilitating the completion of the required procedure without pain and awareness of the child [14]. Targeted depth of sedation, the health status of the child such as fasting state and drug property, which is to be administered all tend to play an important part.

Critical aspects of PSA include pre-procedural evaluation of the patients, constant monitoring of the child before, during and after the procedure, proper procedure documentation, equipment as well as the staff needed in addition to the type of medication that is recommended [3]. Various national and international studies done on this topic have reported evaluating these outcomes in pediatric departments, but specifically, in oncology units, such studies are only a handful [4,7-9].

The objective of this clinical study was to evaluate the safety and efficacy of procedural sedation and analgesia in pediatric oncological patients in our tertiary care hospital.

## Materials and methods

This cross-sectional observational study using a non-probability convenient sampling technique was done at Indus Hospital, Karachi, after approval from the Institutional Review Board. The electronic medical records of children between ages six months and 16 years receiving procedural sedation and analgesia (PSA) for oncological procedures were reviewed over three months from July 2018 to September 2018. This included patients for oncology procedures (lumbar puncture, intrathecal chemotherapy, bone marrow aspiration +/- biopsy). Non-anesthesiologist (intensive care physician/emergency physician certified in Pediatric Advanced Life Support) provided PSA. The American Society of Anesthesiology (ASA) guidelines for PSA was used to assess the patients with inclusion of those with ASA physical status Class I and Class II to whom the administration of PSA was safe. Two drugs used for these procedures included intravenous ketamine in a dose of 0.5-1 mg/kg, followed by intravenous propofol initially in a dose of 1-2 mg/kg. The dose was subsequently titrated as per need during the procedure to keep the patient sedated. Oxygen administration through a face mask and pulse oximeter attachment was done for those recovered in the recovery unit for post-procedure care. Children were excluded from the study if they had received a drug for anxiolysis or analgesia purely without the intention of sedation.

Data collection and analysis

After getting approval from the institutional ethics review committee, demographic features such as age, gender, and other information collected from patients who underwent procedural sedation for procedures such as lumbar puncture alone, bone marrow aspiration alone or both, and occurrence of sedation failure or adverse events were noted. The safety of the drug was described as the administration of a drug that was not associated with any adverse events. Efficacy was defined as the desired effect of medication observed. An adverse event defined as apnea, hypoxia, hallucinations, allergic reactions, or any major events such as cardiac arrest that required cardiopulmonary resuscitation or any case leading to endotracheal intubation. Sedation failure was defined as the inability to achieve adequate sedation with an ideal drug dosage. Data was entered into and analyzed using Statistical Package for Social Science, version 21. Mean and the standard deviation was used for continuous variables like age, the dose of drugs, the total number of patients given procedural sedation, and the total number of procedures (lumbar puncture alone, bone marrow aspiration alone or both lumbar puncture and bone marrow aspiration). Frequency and proportions were used for categorical variables like gender, diagnosis, and type of adverse events. The median and interquartile range was reported for non-Gaussian distributed variables. Simple descriptive statistics were applied. The safety and efficacy of a drug were reported in percentages. 

## Results

A total of 565 children were enrolled in the study with a male preponderance (65.1%). The median age of the patients was 7.4 years (Interquartile range: 4.8-10.9 years). A total of 1216 oncological procedures were performed, out of which lumbar puncture was the commonest procedure performed (n = 956; 78.6%) followed by bone marrow aspirate only (n = 137, 11.3%) and both (n = 123, 10.1%; Table 1).

The majority (82.7%) of the patients were diagnosed with leukemia, 10.4% with lymphoma, 1.9% had sarcoma, and 4.2% had other types of cancer. Furthermore, 4.4% of the children presented with blood-related diagnoses (Table 1).

**Table 1:** Characteristics of study participants n, sample size; IQR, interquartile range

Variable	n (%)
Age (Years)	
Median (IQR)	7.4 (4.8-10.9)
Min – Max	0.83-16
Gender	
Male	368 (65.1)
Female	197 (34.9)
Total	565 (100)
Procedure	
Bone marrow aspiration only	137 (11.3)
Lumbar puncture only	956 (78.6)
Bone marrow aspiration and lumbar puncture both	123 (10.1)
Diagnosis	
Leukemia	467 (82.7)
Lymphoma	59 (10.4)
Sarcoma	11 (1.9)
Other cancer	24 (4.2)
Blood-related	25 (4.4)
Propofol Doses (mg/kg)	
1.0	675 (55.5)
1.5	292 (24)
2.0	162 (13.3)
2.5	58 (4.8)
3.0	17 (1.4)
3.5	7 (0.6)
4.0	5 (0.4)
Propofol Doses (mg/kg)	
Median (IQR)	1 (1-1.5)
Min – Max	1-4
Adverse events	
Hypoxia	8 (0.7)
None	1208 (99.3)
Total	1216 (100)

In majority of the procedures (n = 675, 55.5%), propofol 1 mg/kg was enough for sedation, whereas in 454 (37.3%) and 87 (7.2%) of the procedures, propofol 1.5-2 mg/kg and 2.5-4 mg/kg were utilized for sedation purposes, respectively (Table 1). The median propofol dosage utilized for sedation was 1 mg/kg (interquartile range: 1-1.5 mg/kg; Table 1).

Only eight (0.7%) of the patients were found to have transient hypoxia as an adverse effect of propofol-ketamine drug with 50% procedures utilizing propofol 1 mg/kg for sedation. Additionally, our results revealed that in procedures where the lumbar puncture was done alone, a significantly lower dosage of propofol was utilized for sedation in comparison to combined procedures and bone marrow aspiration alone (Median, Interquartile range: 1 [1-1.5]; 2 [1.5-2.5]; and 2 [1.5-2.5], p = 0.000 respectively; Table 2).

**Table 2:** Propofol levels in procedures (bone marrow aspiration, lumbar puncture or both) Results are based on two-sided tests with a significance of level 0.05. For each significant pair, the key of the category with the larger median (a,b,c) appears under the category with the smaller median. *P-value <0.05, **P-value <0.0001, ¶ Kruskal–Wallis test n, sample size; IQR, interquartile range a, bone marrow aspiration only; b, lumbar puncture only; c, lumbar puncture and bone marrow aspiration both

	Propofol Doses (mg/kg)	P-value
(Bone marrow aspiration only)^a^, n=137		0.000**¶
Mean ± Standard deviation	2 ± 0.6	
Median (IQR)	2 (1.5-2.5)	
Min – Max	1-4	
(Lumber puncture only)^b^, n=956		
Mean ± Standard deviation	1.2 ± 0.3	
Med (IQR)	1 (1-1.5) ^a,c^	
Min – Max	1-4	
(Lumbar puncture and bone marrow aspiration both)^c^, n=123		
Mean ± Standard deviation	2.1 ± 0.6	
Med (IQR)	2 (1.5-2.5)	
Min – Max	1-4	

## Discussion

This study was done to find out the safety and efficacy of procedural sedation and analgesia in pediatric oncological patients. Ketamine and propofol were the two medications used for evaluation of their safety and efficacy. The combination of these two drugs proved to be efficacious as the desired effect was achieved in all patients and safe as just 8 (0.7%) patients experienced minor adverse effects (hypoxia), and none of them had any major complication. Ketamine has its anesthetic, analgesic, and amnestic properties, and propofol is a hypnotic agent preferred for such short procedures as it is short-acting and does not accumulate with multiple doses.

A study conducted by Kuppenheimer *et al*. mentioned that propofol is linked with fewer adverse effects and a short recovery period [15]. The evaluation of 1216 procedures included in our study showed safety and efficacy comparable to another study conducted by Haque *et al*., but the range of procedures assessed in our study is much larger [8]. In this study, we observed that a propofol dose of 1 mg/kg was enough for most patients in contrast to other studies in which higher doses were used [8-9]. There are other safe combinations like Mahajan *et al*. suggested other combinations such as fentanyl and midazolam which are considered a safe regimen in children [16]. A study by Arora *et al*. discussed the common and safe practices of ketamine and propofol [13]. The most common procedure was a lumbar puncture consistent with the findings of a previous study [8].

The median age of the patients was 7.4 years compared to another study, with a median age of 4.2 years [8]. The majority of the patients were males. The procedures were done by non-anesthetics, as the patients who undergo conscious or procedural sedation have preserved airway reflexes, so the use of anesthetic may be unnecessary. However, these non-anesthetics trained in pediatric advanced life support (PALS) could anticipate or handle any encountered airway emergency [10]. Presently, we the term procedural sedation instead of conscious sedation.

In contrast to our study, Bhatt *et al*. reported an overall frequency of adverse events in about 11.7% of patients with oxygen desaturation in 5.6% and vomiting in 5.2% of patients [17]. The reported incidence of adverse events in patients given ketamine alone was 0.4%, which was the lowest. Propofol alone and in combination with ketamine recorded around 2.1% complications. The study proposed that preprocedural antiemetic use can reduce the odds of vomiting by as high as 50%. The use of preprocedural opioids is strongly associated with an increased risk of an adverse event regardless of the sedation drug used [17]. A study conducted by Crea *et al*. compared the incidence of adverse effects caused by propofol and alfentanil with propofol and ketamine, and their study findings suggested that although there are some adverse effects associated with propofol and ketamine, their use is much safer than the other combination and recommended its use in these patients [18].

Ketamine is the most commonly used agent for sedation in painful procedures due to its potent analgesic and amnesic effect, in addition to minimal circulatory or respiratory depression [19]. A relative sparing of effects on protective airway reflexes makes ketamine useful, especially for non-fasting procedures [20]. The increase in blood pressure and pulse associated with ketamine administration maintains the cardiac output; however, in critically ill patients having depleted catecholamine, ketamine can lead to marked hypotension or bradycardia and even cardiac arrest [21]. However, the use of propofol as a bolus has been practiced for short procedural sedation, while continuous infusions are used in prolonged motionless sedation [22]. Since propofol has no analgesic effect and has an only sedative effect, therefore, it is mostly used in adjunction with other potent analgesics such as ketamine or fentanyl, especially in painful procedures [23]. 

This study provides a detailed review regarding the administration of ketamine and propofol, two drugs that can be used to provide procedural sedation and analgesia with minimal adverse effects in children undergoing oncology procedures and their regular use with adequate dose may lead to an improvement in overall treatment compliance by reducing associated pain and anxiety.

## Conclusions

According to the results of our study, the majority of the pediatric patients reported no adverse events during the procedure with ketamine and propofol. Therefore, we conclude that both ketamine and propofol were both safe and efficacious as both sedative and analgesic in procedures on pediatric oncology patients.
